# Determinants of frequency and longevity of hospital encounters' data use

**DOI:** 10.1186/1472-6947-10-15

**Published:** 2010-03-16

**Authors:** Ricardo J Cruz-Correia, Jeremy C Wyatt, Mario Dinis-Ribeiro, Altamiro Costa-Pereira

**Affiliations:** 1 Department of Biostatistics and Medical Informatics, Faculty of Medicine of University of Porto, Al Prof Hernâni Monteiro, 4200-319 Porto, Portugal; 2 Centre for Research in Health Technologies and Information Systems - CINTESIS (Centro de Investigação em Tecnologias e Sistemas de Informação em Saúde), Faculty of Medicine of University of Porto, Al. Prof. Hernâni Monteiro, 4200-319 Porto, Portugal; 3 Health Informatics Centre, University of Dundee, Dundee, UK

## Abstract

**Background:**

The identification of clinically relevant information enables improvement in user interfaces and in data management. However, it is difficult to identify what information is important in daily clinical care, and what is used occasionally. This study aims to determine for how long clinical documents are used in a Hospital Information System (HIS).

**Methods:**

The access logs of 3 years of usage of a HIS were analysed concerning report departmental source, type of hospital encounter, and inpatient encounter ICD-9-CM main diagnosis. Reports median life indicates the median time elapsed between information creation and its usage. The models that better explains report views over time were explored.

**Results:**

The number of report views in the study period was 656 583. Fifty two percent of the reports viewed by medical doctors in emergency encounters were from previous encounters - 21% at outpatient attendance, 19% in inpatient (wards) and 12% during emergency encounters. In an inpatient setting, 20% of the reports viewed were produced in previous encounters. The median life of information in documents is 1.5 days for emergency, 4.8 days for inpatient and 37.8 days for outpatient encounters. Immune-haemotherapy reports reach their median lives faster (7 days) than clinical pathology (15 days), gastroenterology (80 days) and pathology (118 days). The median life of reports produced in inpatient encounters varied from 36 days for neoplasms as the main diagnosis to 0.7 days for injury and poisoning. The model with the best fit (R^2 ^> 0.9) was the exponential.

**Conclusions:**

The usage of past patient information varied significantly according to patient age, type of information, type of hospital encounter and medical cause (main diagnosis) for the encounter. The exponential model is a good fit to model how the reports are seen over time, so the design of user interfaces and repository management algorithms should take it in consideration.

## Background

The age of data is one of the factors usually used to assess data relevance, typically making new information more relevant to the current search. As an example, data that were at least three days old has been categorized as "old data" in the emergency setting [1]. We argue that data ages are likely to differ according to its type, i.e., some clinical reports are less ephemeral than others and so are found useful longer than others. Also, the context of healthcare (e.g.: hospital environment, primary care, oncology), health conditions and patient age probably influences the way information maintains its relevance [2].

In this study we aimed to determine for how long clinical documents are used by health professionals in a Hospital Information System considering the setting of information request and its content.

The practice of medicine has been described as being dominated by how well information is collected, processed, retrieved, and communicated [3]. Patient records, the patient and published evidence are the three information sources needed to practice evidence-based medicine [4], being complemented with the health professional own experience in daily routine.

There is a great demand to create efficient integrated electronic patient records to facilitate the communication process between health professionals by delivering in a single interface data from many different information systems [7]. These systems are evolving to meet people's needs by implementing larger networks, allowing patient access and integration of ever more items of patient data [8]. Although great advances have been made over the years, on-demand access to clinical information is still inadequate in many settings, contributing to duplication of effort, excess costs, adverse events, reduced efficiency [9] and inability to take full advantage of existing IS [10]. Although it is widely accepted that full access to integrated electronic patient records and instant access to up-to-date medical knowledge significantly reduces faulty decision making resulting from lack of information [5,11,12], there is still very little evidence that life-long EHR improve patient care [13]. Shapiro et al. found that, although emergency department doctors believe their patients would benefit from longitudinal records, they only try to obtain such data in 10% of the cases [14]. Recently Hripcsak et al. [1], described the access rates to WebCIS in the emergency department. The results indicate that data generated before the current emergency visit are accessed often, but not a majority of the times, even when the user is notified of the availability of data.

Jones showed that viewing lab results within the context of the patient record made physicians alter their ordering patterns accordingly [15]. Distinguishing between relevant and useless information enables enhanced graphical user interfaces (GUI) to highlight the most relevant information, as well as improved data management by choosing storage devices with better performance for relevant data. However, it is hard to understand what information is really important to daily clinical care, and what is simply occasionally used [16].

Between May 2003 and May 2004, the Department of Biostatistics and Medical Informatics implemented a Virtual Patient Record (VPR) [17] for the Hospital S. João (HSJ), a tertiary university hospital with over 1,350 beds that is the main referring hospital in the north region of Portugal for all medical and surgical specialties.

The system integrates clinical data from 12 legacy Departmental Information Systems (DIS) and the Diagnosis Related Groups and Hospital Administrative databases, aiming to deliver the maximum information possible to health professionals. Over 1,300 medical doctors use the system on a daily basis and the HSJ-VEPR retrieves an average of 3,000 new reports each day [17,18], adding up to more than 3 million reports collected so far.

**Figure 1:**
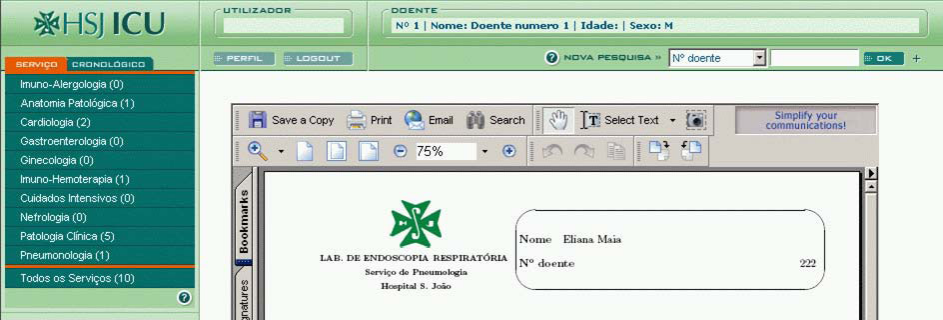
**Viewing module interface of the VPR showing an example of a requested clinical report in PDF format**.

The main components of the VPR are a web-interface (see Figure 1), an integration system and a central repository. The web-interface was designed to include graphical components and layouts to summarise past patient data (patient chronological bars), and folders that reproduce the traditional types of patient record organisations (source, chronological and problem views). It allows ubiquitous access to heterogeneous data sources. The VPR was made available for testing in October 2004, and since December 2004 it started to be known and routinely used.

The authors of this paper have done some preliminary studies aiming to answer for how long are clinical documents useful for health professionals in a hospital environment considering its' content and the context of information request. Our results show that some clinical reports are still used after one year regardless of the context in which they were created, although significant differences exist in reports created in distinct encounter types. The median life of reports by encounter type is 1.7 days for emergency, 3.9 days for inpatient and 27.7 for outpatient encounters. We concluded that the usage of patients past information (data from previous hospital encounters), varied significantly according to the setting of healthcare and content [19].

In this paper we extend our previous work to include the study of associations between the visualization of clinical reports and patient characteristics (e.g. age), the source of the reports, the type of hospital encounter and the main diagnosis for the hospitalization. Also, a mathematical model on how the reports are seen over time is explored.

## Methods

### Participants

This study focuses on sessions and report viewings in the VPR from the last quarter of 2004 to the end of 2007 in HSJ. From the last quarter of 2004 until the end of 2007, the hospital had about 1,583,000 outpatient encounters, 200,000 day-care encounters, 770,000 emergency encounters, 135,000 inpatient encounters and 72,000 emergency consultations adding up to more than 2,760,000 encounters. 977,800 new reports referring to 221,224 distinct patients were collected by the VPR in the same period.

### Ethical approval

The Health Ethical Commission of the HSJ approved this study (Comissão de Ética de Saúde do HSJ), having the reference number 45/2010.

### Procedures for data collection and preparation

The data needed for this study was retrieved from three different Oracle database schemas: (1) the VPR patient database, which included patient identification and the list of clinical documents integrated; (2) the access logs including sessions, health professionals' identification and category and document views; (3) and a hospital encounters database that includes patient identification and the list of encounters since 1993. All sessions from the development team (n = 2,918) in the VPR were excluded from this analysis.

Report views that did not take more than three seconds were considered mistaken views and excluded from this analysis. The VPR is not capable of directly measuring how long reports are viewed, so it was calculated the maximum time of viewing based on the difference between the report request and the following user request on the same session. The number of mistaken views found was 14,972 and represents 2.2% of all views.

Some DIS (especially laboratory IS) send several versions of the same report as new findings occur. In some analysis these different report versions are grouped together to be considered as one single report regardless of version (grouped versions); in others analysis they are considered separately as distinct reports (all versions). The grouped versions method allows to study when does the information inside reports is accessed regardless of the report version, whilst the all versions method allows to study when are files accessed.

### Variables

#### Users

All logins are staff numbers generated by the human resources department. In this analysis, the users are identified by their logins.

#### Computers

In the network of the hospital each IP number is associated with the network card's MAC address. In this analysis, each computer is identified by its' network IP number.

#### Clinical report median life

The clinical report median life is the time it takes to occur half of the views by the users. It is estimated by calculating the median report age (difference between the maximum date of the view and the date of making the report available) within a set of views.

#### Definition of type of encounter

The VPR system does not know in what context (inpatient, outpatient or emergency) the user is viewing each report. The context was inferred by comparing the date of view and the dates of the different patient encounters. When the date of view matches an encounter, that encounter is associated with the report viewing. When no match is made no assumption is made regarding the encounter.

*Concomitant views *refer to cases when the medical doctor is viewing a report generated on the present patient encounter (e.g. a doctor asks for a clinical pathology lab exam for an inpatient and the results are seen during that inpatient encounter). *Last views *refer to cases when the doctor is viewing a report produced on the last encounter whatever its type (e.g. a doctor requests for a imuno-hemotherapy lab exam on an outpatient consultation, and views it in the following outpatient consultation). *Previous views *refer to views of reports produced previous to the last encounter (e.g. in an emergency encounter the doctor views a report produced 4 encounters ago in an outpatient consultation).

### VPR usage analysis

#### Usage evolution

The records were grouped by time periods (quarters) of the date of session start. The *views *variable refers to all versions of the reports. The number of patient encounters is the sum of all inpatient, outpatient, emergency and hospital day-care encounters occurred in the hospital. Distinct users are calculated by counting the number of different logins that occurred in session logs. The views per user per 10 000 encounters is calculated by . Distinct computers are calculated by counting the number of different IP addresses that occurred in session logs.

#### Reports' median half analysis

To study the factors that relate to reports' median life, the following variables were studied: patient sex and gender, type of report, hospital encounter related to the report, referral IS and admission and discharge diagnosis in inpatient encounters.

#### Patient age and gender

Patients are considered as children if their age is 0 to 12 years old, teenager if 13 to 19 years old; young adult if 20 to 34 years old; adult if 35 to 54 years old; old adult if 55 to 69 years old; senior if more than 70 years old). In this case the median life of the grouped versions of the reports was considered.

#### Type of report

The type of report is determined by the DIS that acts as the VPR feeder system. In the case of immune-haemotherapy the VPR can subdivide them into molecular biology, haemostasis, transfusion laboratory and viral markers since September 2006. In this case the grouped versions of the reports were considered.

#### Type of hospital encounter

Each report retrieved may be associated with a patient encounter that can be of the following types: inpatient, outpatient, emergency, day-care, radiology and lab result. In this case the grouped versions of the reports were considered, either in the ration of reports viewed and in the median life and dispersion ratio.

#### Hospital inpatients encounter main diagnosis

The International Classifications of Diseases, 9^th ^revision, Clinical Modification (ICD-9-CM) is based on the official version of the World Health Organisation's 9^th ^Revision, International Classifications of Diseases (ICD-9). ICD-9 is designed for the classification of morbidity and mortality information for statistical purposes and for indexing of hospital records by disease and operations, for data storage and retrieval. In Portuguese hospitals, each inpatient encounter has an ICD-9-CM diagnosis code associated as the main diagnosis of the inpatient encounter. These codes are classified in multi-level hierarchy, and are grouped in chapters, sections and categories. In this analysis the median lives of grouped versions of reports retrieved in inpatient encounters associated with chapters, sections and categories were calculated.

#### Encounter setting analysis

It was also studied the relation between the types of hospital encounters associated with the creation and view of the reports. This portion of the analysis only takes in consideration visualizations from 2005 and 2006 due to difficulties in accessing detailed data from hospital patient encounters of 2007. All versions of the reports were considered in this analysis.

#### Median life of reports mathematical model

To obtain a mathematical model, we studied all report views occurring during a 4 months period (September to December 2007). This included reports created before September 2007 and during the studied period. For each of these months the views were grouped according to the age of the reports (e.g. views of report that were one month old, two moths old, and so on, until 44 months old). Then the cumulative frequency was obtained and used to calculate the cumulative percentage of views. These values were then used to obtain logarithmic and exponential trend lines.

## Results

### Usage evolution

**Figure 2:**
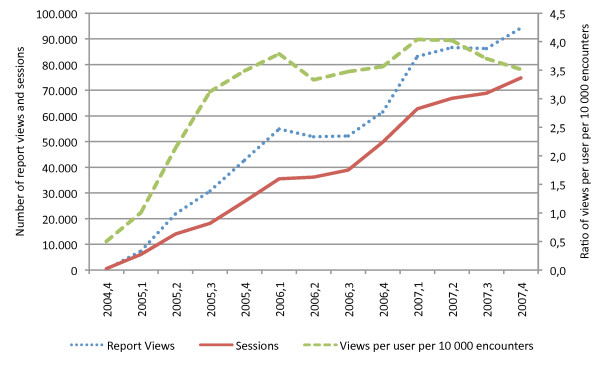
**Chart presentation of VPR evolution per quarter regarding sessions and report views per user per 10,000 encounters**. Table with evolution of VPR usage namely: user sessions, report viewings, distinct users and distinct computers (PCs).

From the 4th quarter of 2004 until the end of 2007 a total of 2,943,294 reports were collected adding up to 970,856 when the different versions of the same report are grouped together. The number of sessions (solid red line) and report views (blue dotted line) has been growing steadily since then (Figure 2). The number of sessions increased 147% in 2006, and 70% in 2007. The number of doctors in the hospital was 1277 in 2005, 1259 in 2006 and 1311 in 2007; the number of distinct users was 616 (48%) in 2005, 794 (63%) in 2006 and 1124 (86%) in 2007. The number of users had an annual growth of 29% users in 2006 and 41% in 2007. In the 4^th ^quarter of 2007, 1.24 reports were viewed per session, 0.4 reports were viewed per patient encounter and 82.4 reports were viewed per user. Also the use of the VPR is more widespread by the hospital computers (975 computers in 4^th ^quarter of 2007). Table 1 also shows the number of report views per quarter of report creation (first column of the table) and per quarter of report view (heading line of table). The last quarter of 2007 has the highest number (n = 36,171) and percentage (39%) of report views of previous quarters ever; in this quarter a large number of reports collected in 2004 (n = 855) are still accessed.

**Table 1:** Crosstab of number of views per quarter of report creation and quarter of report view, and ratio of report views of regarding reports collected in previous quarters.

Time interval (quarter)		Quarter of report view													Total	
				
		2004	2005				2006				2007					
							
		4^th^	1^st^	2^nd^	3^rd^	4^th^	1^st^	2^nd^	3^rd^	4^th^	1^st^	2^nd^	3^rd^	4^th^	Views	Reports
2004	1^st^	33				1		1				1			**36**	**60**
	2^nd^	8	120	348	220	194	228	186	160	177	160	150	127	186	**2 264**	**96 944**
	3^rd^	17	273	504	440	460	440	206	209	185	253	302	254	296	**3 839**	**136 217**
	4^th^	219	710	981	670	646	848	437	338	385	521	500	469	433	**7 157**	**142 366**

2005	1^st ^*		5 930	5 235	2 831	3 069	2 860	2 131	1 411	1 588	2 121	2 022	1 539	1 682	**32 419**	**219 691**
	2^nd^			14 045	5 714	2 529	2 503	1 260	921	865	1 168	1 092	902	941	**31 940**	**195 047**
	3^rd^				18 328	7 484	3 469	1 721	1 104	839	1 050	954	815	942	**36 706**	**191 002**
	4^th^					27 293	9 405	2 583	1 524	1 282	1 515	1 138	1 088	1 274	**47 102**	**200 256**

2006	1^st^						33 782	8 165	2 797	2 080	2 427	1 584	1 246	1 441	**53 522**	**221 930**
	2^nd^							33 670	8 572	3 291	2 924	2 032	1 475	1 477	**53 441**	**224 021**
	3^rd^								33 890	8 792	4 277	2 215	1 833	1 769	**52 776**	**239 974**
	4^th^									41 062	11 622	4 018	2 455	2 297	**61 454**	**244 472**

2007	1^st^										52 978	13 563	4 503	3 134	**74 178**	**265 134**
	2^nd^											55 067	13 492	5 040	**73 599**	**257 098**
	3^rd^												54 317	15 259	**69 576**	**270 213**
	4^th^													56 574	**56 574**	**272 090**

**Total**		**277**	**7 033**	**21 113**	**28 203**	**41 676**	**53 535**	**50 360**	**50 926**	**60 546**	**81 016**	**84 638**	**84 515**	**92 745**	**656 583**	**3 176 515**

**Views of reports of previous quarters**		21%	16%	33%	35%	35%	37%	33%	33%	32%	35%	35%	36%	39%		

* - in the 1^st ^quarter of 2005 many pathology reports were retrieved regarding previous dates

### Reports' median life

#### Patient age and gender

The median life of the reports for men (n = 141,568) is 20.09 days and for women (n = 148,321) is 20.11 days. The percentile 25 in women is higher (2.0 days) than in men (1.2 days). The reports of the children and elderly patients are viewed for less time (median life is 4.1 and 6.7 days respectively) than the reports of the other groups (median life is 15.9, 24.0, 28.9 and 20 for teenagers, adults and seniors).

#### Report departmental source

The distribution of report views from each DIS (type of report) is shown in Table 2.

**Table 2:** Number of grouped versions views, their median life, percentiles 25 and 75 (in days) per type of report, type of encounter associated with report creation, and according to chapter of main inpatient ICD-9-CM.

Determinant	Number of views	Median life	Percentiles	
			
			p25	p75
**Report departmental source**				
*Pathology*	165 899	118	28	346
*Gastroenterology*	18 616	80	13	308
*Gynaecology*	962	74	35	220
*Obstetrics*	2 382	37	2	216
*Pneumology*	5 057	26	6	118
*Intensive care*	1 583	20	2	106
*Cardiothoracic*	3 317	16	4	49
*Clinical pathology*	191 463	15	1	54
*Paediatric gastroenterology*	385	14	3	46
*Immune-haemotherapy*	266 631	7	0.4	53
Molecular biology*	9 142	49	21	121
Viral markers*	18 557	25	8	81
Transfusion laboratory*	3 615	3	0.9	20
Haemostasis*	52 958	1	0.1	13

**Type of hospital encounter**				

*Lab results*	1 340	56.5	8.5	234.3
*Outpatient*	130 946	37.8	12.9	149.2
*Day-care*	1 091	18.2	4.9	68.0
*Inpatient*	108 737	4.8	0.4	67.6
*Emergency*	39 884	1.5	0.1	29.0

**Chapter of main inpatient ICD-9 CM diagnosis**				

*Neoplasms*	21 097	36.0	1.8	268
*Endocrine, nutritional and metabolic diseases, and immunity disorders*	2 503	26.2	1.1	211
*Diseases of the genitourinary system*	3 837	26.1	1.2	156
*Symptoms, signs, and ill-defined conditions*	817	16.0	1.0	147
*Diseases of the digestive system*	11 308	11.9	0.9	98
*Infectious and parasitic diseases*	7 842	11.1	0.8	85
*Complications of pregnancy, childbirth, and the puerperium*	4 211	10.7	0.8	106
*Diseases of the skin and subcutaneous tissue*	693	10.3	1.0	119
*Diseases of the blood and blood-forming organs*	1 163	8.0	0.9	77
*Mental disorders*	385	7.9	1.1	63
*Certain conditions originating in the perinatal period*	122	7.0	0.9	109
*Diseases of the respiratory system*	5 154	5.1	0.7	63
*Diseases of the nervous system and sense organs*	2 076	4.8	0.8	47
*Diseases of the musculoskeletal system and connective tissue*	2 418	3.8	0.7	70
*Supplementary classification of factors influencing health status and contact with health services*	3 160	1.6	0.8	18
*Congenital anomalies*	1 182	0.8	0.1	30
*Diseases of the circulatory system*	20 665	0.8	0.1	11
*Injury and poisoning*	7 006	0.7	0.1	6

* - the subdivision of the immune-haemotherapy reports is only possible in the last months of 2006 and in 2007, which explains why the sum of the four sub-divisions is lower the total of immune-haemotherapy reports

The lab reports (immune-haemotherapy, clinical pathology and pathology) represent almost all (96%) of the report views in the system. Although pathology and immune-haemotherapy reports are only digitally available for hospital users through the VPR, only about 65% of them were actually accessed at least once in 2007. The clinical pathology reports are also available through other applications in the Hospital.

The reports from immune-haemotherapy reach their median life in 7 days. Reports from paediatric gastroenterology, clinical pathology and cardiothoracic have similar behaviour, having their median life reached at 14, 15 and 16 days respectively. Reports from pathology and gastroenterology have their median life reached after 118 and 80 days respectively.

#### Type of hospital encounter

Table 2 also shows the median life results grouped by type of hospital encounter. Reports requested in encounters whose objective is to accomplish lab analysis, have longer median lives (56.5 days); the median life decreases to 37.8 days in outpatient and 18.2 in day-care visits; inpatient and emergency encounters are the group with lowest median lives (4.8 and 1.5 days respectively).

Most of the views (percentile 75) of reports generated in emergency encounters are viewed in the first month (29 days); this value increases to two months in inpatient and day-care encounters; and to more than 5 months in outpatient and lab results encounters.

#### Hospital inpatient encounter main diagnosis

Table 2 shows the number of views, the median lives and percentiles 25 and 75 of reports created in inpatient encounters grouped by ICD-9-CM chapters. Reports associated with neoplasms encounters have their median lives in 36 days and 75% of the views occur in the first 268 days. Also reports related to endocrine, nutritional and metabolic diseases, and immunity disorders and diseases of the genitourinary system have high median lives (more than 26 days).

On the other end, reports related with injury and poisoning encounters have their median lives in 0.7 days ≈ 17 hours and 75% of the views occur in the first 6 days. Reports related to diseases of the circulatory system and congenital anomalies also have low median lives (0.8 days ≈ 19 hours).

There are 118 ICD-9-CM sections. Only sections with more than 20 cases (n = 100) were considered for further analysis. Various types of reports related to neoplasms encounters have high median lives, namely when coded with the sections *carcinoma in situ *(356 days), *malignant neoplasm of bone, connective tissue, skin, and breast *(156.3 days) and *malignant neoplasm of genitourinary organs *(134.1 days); on the other hand sections like *malignant neoplasm of lymphatic and haematopoietic tissue *(2.9 days) or *neoplasms of unspecified nature *(13.7 days) have lower median lives. The median lives of reports related to the sections of *injury and poisoning *chapter range from 3.1 days of section *certain traumatic complications and unspecified injuries *and 0.3 days ≈ 7.2 hours of *injury to blood vessels*.

#### Setting of encounter

Table 3 shows the proportion of visualizations by setting at visualization according to the type of encounter associated with the production of the report.

**Table 3:** Percentage of views grouped by setting of visualization and report creation (n = 193,230) in 2005-2006

	Encounter of report creation (%)								
		
	*Present*	*Last*				*Any Previous*			
								
Viewed in		Emergency	Inpatient	Outpatient	Total	Emergency	Inpatient	Outpatient	Total
*Emergency *(n = 7939)	48	5	2	3	10	7	17	18	42
*Inpatient *(n = 97382)	64	12	0	1	13	3	10	10	23
*Outpatient *(n = 87909)	0	1	3	22	26	3	13	57	73

In emergency encounters, there is a balance between the information produced in the current encounter (48%) and in last and previous ones (52%). Reports from outpatient (18%) and inpatient (17%) visits are accessed more after the last encounter than emergency visits (7%).

Most views in inpatient encounters are of information produced in the current encounter (64%). Reports generated in the last emergency encounter are also important to the current inpatient encounter (12%). Older inpatient (10%) and outpatient (10%) reports are still accessed in new inpatient encounters.

By contrast, in outpatient encounters health professionals rarely refer to information produced in the concomitant encounter (near 0%) and are generally associated with last (22%) or previous (57%) outpatient encounters. Previous inpatient encounters reports are also used in the outpatient context (13%).

**Figure 3:**
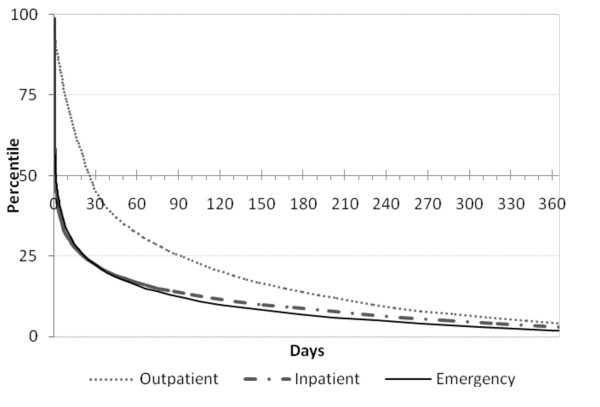
**Chart representing the speed of aging of the reports produced in outpatient, inpatient and emergency encounters; the median lives are the values in percentile 50**.

Figure 3 illustrates the report median life when produced in inpatient, emergency or outpatient encounters. It shows that outpatient reports age slower than inpatient and emergency reports. Nevertheless a few reports of each encounter type are still accessed after one year.

#### Median life of reports mathematical model

Table 4 presents the logarithmic and exponential regression equations and the corresponding R^2 ^value for the report visualizations of 4 different months. X represents the current age of the report in days, and y is the cumulative percentage of all report views on each considered month of 2007. The exponential fit was better as its R^2 ^values are higher (0.908 in average) that the logarithmic (0.869 in average), and the number of R^2 ^values higher than 0.9 is greater (4 out of 4) than in the logarithmic (1 out of 4). The R^2 ^values of the exponential regression for each month regarding the (September to December 1997) are 0.904, 0.904, 0.905 and 0.919.

**Table 4:** Exponential and logarithmic regressions of median life of reports viewed between September and December 2007.

Month of 2007	Exponential		Logarithmic	
		
	Equation	R^2^	Equation	R^2^
December	y = 0.5826e^-0.12x^	0.904	y = -0.161 × ln(x) + 0.554	0.842
November	y = 0.6287e^-0.131x^	0.904	y = -0.165 × ln(x) + 0.563	0.850
October	y = 0.7159e^-0.11x^	0.905	y = -0.176 × ln(x) + 0.628	0.922
September	y = 0.5975e^-0.125x^	0.919	y = -0.168 × ln(x) + 0.572	0.863

X represents the current age of the report (in days), and y is the cumulative percentage of all report views on each considered month of 2007.

## Discussion

Although the median life of both genders is very similar (20.09 and 20.11 days for men and women), 25% percent of the men reports are seen in the first day whilst it takes two days for women reports to get to the same percentage. Reports tend to have higher median lives as the patients' age is nearer of middle age (40 years). These results are probably related to the most common diseases in each gender and age categories and the period patients are followed in hospital encounters.

Report median lives are very different depending on the departmental source and report content. They range from 1 day of haemostasis reports (percentile 75 is 13 days) to 118 days of pathology reports (percentile 75 is 346 days). These results clearly show that different reports are needed faster (e.g. percentile 25 of haemostasis is 0.1 days ≈ 2 hours) than others (e.g. percentile 25 of gynaecology is 35 days). It also shows that some reports are needed for longer periods of time (e.g. percentile 75 of pathology is 346) than others (e.g. percentile 75 of haemostasis is 13 days).

The analysis of report median lives according to ICD-9-CM shows a close relation between report usefulness along the time and the main diagnosis related to each inpatient encounter; the median life of neoplasms (median life = 36 days; p25 = 1.8; p75 = 268) is 51 times higher than injury and poisoning (median life = 0.7 days; p25 = 0.1; p75 = 6). The main diagnosis of the inpatient encounter certainly is associated with the possibility of happening future encounters within the same hospital (e.g. chronic diseases and neoplasms are followed for longer periods than injuries or poisoning). Unfortunately in this scenario, it is not available a similar diagnosis code describing the reason for encounter in outpatient, emergency and day-care encounters.

Two distinct phases can be defined in inpatient and emergency encounters: firstly an initial patient observation and evaluation based on past and current information help to achieve a diagnosis; secondly follows a sequence of iterations of the diagnostic-therapeutic cycles in which after each therapeutic action new observations are made (e.g. lab analysis) to monitor the patient state [20]. The main difference between both phases is that in the first phase there is a more active use of information collected in previous encounters. The longer the encounter (e.g. inpatient are normally longer than emergency encounters) more iterations occur in the second phase resulting in more views of concomitant encounter reports (64% in inpatient versus 47% in emergency) than previous reports.

However, the large number of views in emergency encounters referring to old reports (53% of all report views) should be stressed as these results probably show a higher use of past data in our case, than on other published studies (5% to 20% of all emergency encounters in Hripcsak et al. [1], and 10% of all emergency encounters in Shapiro et al. [14]). Moreover, these reports were initially produced in different encounter types, which illustrate the importance of longitudinal electronic health records even in emergency scenarios.

It is important to note that most of these reports when viewed in a new encounter, gain a second life after their initially planned life cycle that was limited to the encounter where it was produced. This fact illustrates how hard is to define what information is really important to clinical care, and what is simply occasionally useful. In the outpatient scenario, one could expect report views to show more clearly a long-term problem-driven patient healthcare. But the number of views if previous emergency and inpatient encounter, shows the importance to medical specialty encounters of unplanned past events.

Regarding the mathematical model, the high values of R^2 ^(> 0.9) shows that the exponential regression is a good fit to model how the reports are seen over time. This model can be used to estimate the relevancy of each report in a particular moment.

Overall, our results show that in the VPR system past patient information is used by doctors in everyday healthcare. Like in Hripscsak et al. this proves that when doctors have access to past information, even if it was collected for a different purpose, they use it to make a diagnosis [1].

### Limitations

The limitations of our study include the fact that each health professionals may access clinical information either by reading the paper patient record, using the VPR or using other IS available on the hospital. We feel that the data sources used are more appropriate to raise hypothesis about how to calculate median life of clinical reports and its' information than to test them.

### Future work

The availability of patient information in integrated clinical Information Systems raises a new kind of problem - too much data, and too little time to select and read it. These systems are collecting hundreds of reports per patient and presenting them for the user to decide which he will select to read. Helping the health professional filter the reports may be the difference between finding relevant information or not. Report usefulness (taking into consideration their median life) varies between 7 and 118 days depending on the department that originated that information. We feel that these variations should have consequences on information presentation and management, instead of pre-determining when information gets old. As an example one could adjust the display time window for recent reports to the estimated median life of each report. Our results show that the context of the current encounter, the type of encounter and department where the report was produced are related to the report longevity.

These results will be influenced by how a particular Hospital operates, how the health professionals work, what IS are made available, and if there are paper records at hand. Each IS should carry out a similar analysis of report use logs, and adapt its GUI and data management to it. If a group of reports of Clinical Pathology were produced before the last 15 days, their relevance should probably be downgraded, leaving space for other reports. On the other hand, pathology reports should be kept online in fast digital archives for much longer, due to the fact that many are still accessed after 18 months years.

**Figure 4:**
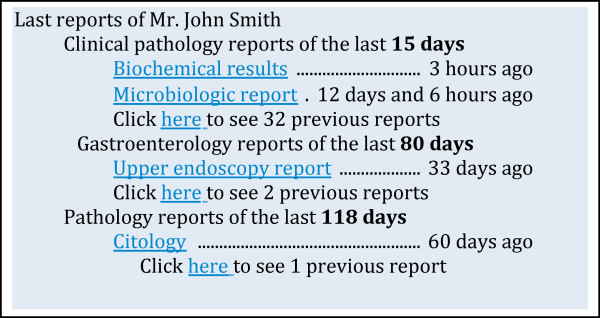
**Example of graphical user interfaces that takes in consideration the median life to highlight most relevant reports**.

Figure 4 shows a prototype GUI that enhances clinical report usefulness by taking into consideration the calculated median life of each report by department. This interface would promote some reports by others according to a calculated time interval for each department, in this case set to the median life (median of previous viewings of same type of reports) of the reports. The authors are implementing interfaces similar to the proposed one and evaluating its usability by health professionals.

## Conclusions

The usage of past patient information in the VPR case-study varies significantly according to patient age, type of information, type of hospital encounter and medical cause (main diagnosis) for the encounter. As more and more patient information is stored, it is very important to efficiently select which one is more likely to be useful and promote it in a scenario where scarcity of resources (screen space, storage space, bandwidth and doctors' time) is very real.

Researchers and developers dealing with the implementation, monitoring and utilization of an integrated Hospital Information System should create mechanisms that automatically take in consideration the usage profiles to efficiently manage available resources.

## Competing interests

The authors declare that they have no competing interests.

## Authors' contributions

RCC was responsible for the study design, organization, data collection, analyses and manuscript preparation. JCW, MDR and ACP advised the study design, and supervised the statistical analysis, the results interpretation and the manuscript preparation. All authors read and approved the final manuscript.

## Pre-publication history

The pre-publication history for this paper can be accessed here:

http://www.biomedcentral.com/1472-6947/10/15/prepub
